# Associations of linear growth and weight gain in the first 2 years with bone mass at 4 years of age in children in Dhaka, Bangladesh

**DOI:** 10.1017/S1368980024002301

**Published:** 2024-11-22

**Authors:** Maimuna Gias, Huma Qamar, Farzana Fariha, Abdullah Al Mahmud, Prakesh Shah, Steven A Abrams, Daniel E Roth, Karen M O’Callaghan

**Affiliations:** 1 Department of Nutritional Sciences, University of Toronto, Toronto, Canada; 2 Centre for Global Child Health, SickKids Research Institute, The Hospital for Sick Children, Toronto, Canada; 3 Nutrition Research Division, International Centre for Diarrhoeal Disease Research, Bangladesh, Dhaka, Bangladesh; 4 Department of Pediatrics, Mount Sinai Hospital, Toronto, Canada; 5 Lunenfeld Tanenbaum Research Institute, Mount Sinai Hospital, Toronto, Canada; 6 Department of Paediatrics and Institute of Health Policy, Management and Evaluation, University of Toronto, Toronto, Canada; 7 Department of Pediatrics, Dell Medical School at the University of Texas at Austin, Austin, TX, USA; 8 Department of Pediatrics, The Hospital for Sick Children, Toronto, Canada; 9 Department of Nutritional Sciences, King’s College London, London, UK

**Keywords:** Bone mass, Linear growth, Weight gain, Child growth

## Abstract

**Objective:**

Growth faltering is widespread in many low- and middle-income countries, but its effects on childhood bone mass accrual are unknown. The objective of this study was to estimate associations between length (conditional length-for-age *z*-scores, cLAZ) and weight (conditional weight-for-age *z*-scores, cWAZ) gain in three age intervals (ages 0–6, 6–12 and 12–24 months) with dual-energy X-ray absorptiometry-derived measures of bone mass (total body less head (TBLH) bone mineral content (BMC), areal bone mineral density (aBMD) and bone area) at 4 years of age.

**Design:**

Associations between interval-specific growth parameters (cLAZ and cWAZ) and bone outcomes were estimated using linear regression models, adjusted for maternal, child and household characteristics.

**Setting:**

Data collection occurred in Dhaka, Bangladesh.

**Participants:**

599 healthy children enrolled in the BONe and mUScle Health in Kids Study.

**Results:**

cLAZ in each age interval was positively associated with TBLH BMC, aBMD and bone area at 4 years; however, associations attenuated towards null upon adjustment for concurrent height-for-age *z*-scores (HAZ) at age 4 years and confounders. cWAZ from 0 to 6 and 6 to 12 months was not associated with bone mass, but every sd increase in cWAZ between 12 and 24 months was associated with greater BMC (7·6 g; 95 % CI: 3·2, 12·0) and aBMD (0·008 g/cm^2^; 95 % CI: 0·003, 0·014) after adjusting for concurrent WAZ, HAZ and confounders.

**Conclusions:**

Associations of linear growth (birth to 2 years) with bone mass at age 4 years were explained by concurrent HAZ. Weight gain in the second year of life may increase bone mass independently of linear growth in settings where growth faltering is common.

Linear growth faltering is a global public health concern among children in low-and-middle income countries due to its association with infectious diseases, mortality and cognitive impairments^(1,2)^. Longitudinal studies in high-income countries have demonstrated associations between linear growth and bone mass accretion, whereby greater gains in length in early life have been associated with a larger bone area in later childhood^(3–6)^. Bone mass tracks longitudinally from childhood to later in life^(7,8)^, such that reduced bone mineral content (BMC) and bone mineral density (BMD) are risk factors for fractures during childhood^(9)^ and osteoporosis later in life^(10,11)^. Characterising associations between growth at specific early-life stages and later bone mass may therefore guide the design of age-specific interventions to promote both length gain and bone mass accrual, which may be particularly relevant to children in low-income settings where undernutrition is widespread.

Long bones are formed through endochondral ossification, which involves calcium accumulation in the primary spongiosa and chondrocyte mineralisation^(12,13)^. Endochondral bone growth reflects an increase in bone length and, under normal physiological conditions, a commensurate increase in bone density^(12)^. Conversely, appositional bone growth involves deposition of bone mineral to the periosteal bone surface thereby increasing bone width (and, consequently, bone area), which ensures stability of growing bones^(14)^. However, the extent to which bone elongation and mineralisation are related in the setting of chronic nutritional deficiencies and growth faltering is uncertain, whereby bone mineral accrual may be influenced by periods of relatively rapid postnatal growth^(15)^.

In a cohort of 4-year-old children in Dhaka, Bangladesh, among whom linear growth faltering is common (22 % with height-for-age *z*-score (HAZ) < –2), we previously found that measures of bone mineral acquisition (i.e. areal bone mineral density (aBMD) and BMC) were low in comparison to a reference from UK-based children of the same chronological age and sex^(16)^. Here, using longitudinal data collected in this cohort since birth^(16,17)^, we aimed to examine the associations of length or weight gain from birth to 2 years of age with dual-energy X-ray absorptiometry (DEXA)-derived bone outcomes (BMC, aBMD and bone area) at 4 years of age.

## Methods

### Study design

This study was based on a secondary analysis of data obtained from the previously completed Maternal Vitamin D for Infant Growth (MDIG) trial (clinicaltrials.gov identifier: NCT01924013) and the follow-up BONe and mUScle Health in Kids study (BONUSKids; NCT03537443). A full description of the methods and primary outcomes for the MDIG trial and BONUSKids study can be found elsewhere^(16–18)^. Briefly, the MDIG trial was a double-blind, randomised placebo-controlled clinical trial of prenatal and postpartum vitamin D_3_ supplementation (*n* 1300) in Dhaka, Bangladesh. The primary outcome was infant linear growth at 1 year of age with further follow-up to 2 years of age^(17)^. The BONUSKids study included a subset of MDIG trial participants (*n* 642) and examined the effects of the prenatal and postpartum intervention on DEXA-derived measure of bone mass, body composition and muscle strength at 4 years of age^(16)^.

### Sample size and eligibility

This study had a fixed maximum sample size based on prior enrolment into the MDIG trial (*n* 1300) and the BONUSKids follow-up study (*n* 642). Inclusion criteria for this secondary analysis mandated the availability of anthropometry measurements (length and/or weight) taken during any of the postnatal timepoints of interest (i.e. 6, 12 and 24 months of age) and a useable DEXA scan at 4 years of age (i.e. scans void of excess motion artifacts). Among the 642 participants enrolled in the BONUSKids study, twenty participants were excluded due to an incomplete DEXA scan, and a further twenty-three participants were excluded due to excess DEXA motion artifacts, resulting in 599 participants who contributed data for the present study.

### Maternal, infant and household characteristics

The last recalled menstrual period and/or ultrasound dating available at the time of enrollment were used to estimate gestational age^(18)^. General health, lifestyle and socio-demographic data were collected at baseline (17–24 weeks’ gestation) through interviewer-administered questionnaires. Socioeconomic status was assessed based on the results from a household survey conducted during the earliest home visit (i.e. one week after enrollment) from which an asset index score was determined using principal components analysis^(19)^. Weekly postnatal visits involved collection of information on breast-feeding practices and consumption of complementary foods during the initial 6 months of life, and the occurrence of diarrhoea was recorded based on caregiver reports; data on dietary patterns or diarrhoea occurrence was not collected at the postnatal visits between 6 and 24 months of age. Mid-gestation maternal hemoglobin (Hb) concentrations were measured at enrollment using a point-of-care hemoglobinometer (Hb 201+, HemoCue, AB).

### Anthropometry

Anthropometric measurements were collected during scheduled postnatal visits using standardised procedures as previously described^(16–18)^. Crown-to-heel infant length was measured using a length board up to 2 years of age (Infant/Child ShorrBoard; Weigh and Measure, Olney, MD, USA and Harpenden infantometer; Holtain, Crymych)^(17,18)^, and standing height was measured at 4 years using a stadiometer (Leicester Height Measure, Chasmors)^(16)^. Weight was measured on a digital scale (Seca 874, Seca)^(16–18)^. The mean of paired anthropometric measurements was used in analyses. Length-for-age *z*-scores (LAZ), HAZ and weight-for-age *z*-scores (WAZ) were derived based on INTERGROWTH-21st or WHO child growth standards, as appropriate^(20–22)^. For this study, we categorised data into four timepoints: earliest measure (<45 d of age), 6 months (182–196 d of age), 12 months (364–416 d of age), 48 months (666–807 d of age) and 4 years (1431–1553 d of age). While the earliest measure was used to maximise the sample size, this timepoint predominantly reflects measurements taken at birth, whereby the median (25^th^, 75^th^ percentile) age at measurement was 1 (1, 3) day.

### Dual-energy X-ray absorptiometry

Total body less head (TBLH; sub-cranial skeleton from base of neck to feet), BMC, aBMD and bone area were examined by DEXA at 4 years of age using a narrow-angle fan-beam DEXA scanner (Lunar Prodigy Advance; GE Healthcare, Madison, WI) in the enhanced analysis mode on enCore software v16·0. Quality of the TBLH DEXA images was assessed independently for alignment and motion artifact, as detailed previously^(16)^. DEXA scans with excessive motion were excluded from analysis. The three densitometry measures reflect different albeit-related measures of bone mass; BMC reflects absolute value of the bone mineral accrued, bone area is a two-dimensional measure of bone size and aBMD represents the mass of BMC per square centimeter of bone. TBLH BMC and aBMD *z*-scores were derived using the Lambda-Mu-Sigma-modelled formulas proposed by Crabtree *et al* ^(23)^.

### Statistical analysis

Histograms, boxplots and kernel density plots were used for visual inspection of the data distributions. All outcome variables (i.e. TBLH BMC, aBMD and bone area) approximated a normal distribution, and hence, transformation was not required. Bivariate relationships between the DEXA-derived bone outcomes and anthropometric variable (LAZ/HAZ or WAZ) at each timepoint were examined using scatterplots with locally weighted scatterplot smoothing.

A conditional growth modelling approach^(24,25)^ was used to assess the associations of interval-specific growth measurements from age 0 to 24 months with each bone outcome (TBLH BMC, TBLH aBMD or TBLH bone area) at 4 years of age. At the first stage, the anthropometric *z*-score measurement (i.e. LAZ or WAZ) at one age timepoint was regressed on the preceding measure to generate standardised residuals, which reflected the extent to which an individual participant grew (i.e. slower or faster) relative to peers within each of three age intervals: earliest measure to 6 months, 6–12 months and 12–24 months of age. Specifically, the conditional length-for-age-*z*-score (cLAZ) for each age interval was generated by deriving the individual participant residual from the simple linear regression of LAZ at the end of an age interval on LAZ at the beginning of an age interval. Conditional weight-for-age-*z*-scores (cWAZ) for each age interval were similarly generated by regressing end-of interval WAZ on start-of-interval WAZ. Conditional *z*-scores may therefore be interpreted as each child’s relative length/weight gain during a specified interval, compared with peers, accounting for the child’s own length/weight at the beginning of the interval.

At the second stage, all cLAZ (or cWAZ) measures were incorporated as covariates in a single regression model for each bone outcome, such that each regression coefficient reflected the effect of growth on each bone parameter in one age interval, independent of growth in the preceding intervals^(26)^. Effect estimates were expressed as the difference in bone outcome per unit increase in growth parameter, with 95 % confidence intervals (CI). *P* < 0·05 was considered statistically significant.

**Figure. 1:**
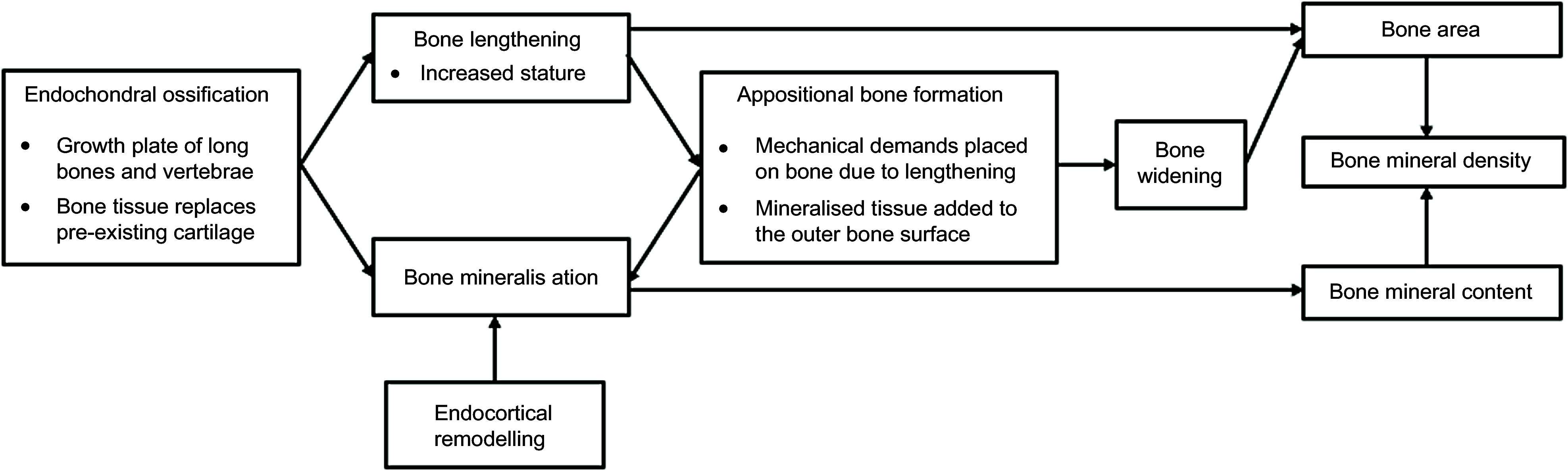
Hypothesised mechanisms by which concurrent HAZ at 4 years and bone parameters at 4 years are coupled. Endochondral ossification promotes bone lengthening and bone mineralisation. The associations of weight gain during the 12–24-month period and increased bone mass (bone mineral content and density), but not bone area, are likely explained by appositional bone formation or endocortical remodelling, rather than endochondral ossification which couples bone lengthening and bone mineralisation.

We first estimated bivariate associations of each conditional growth parameter (i.e. cLAZ for each age interval) with each bone outcome (referred to as ‘base models’). Then, models were expanded to adjust for a set of covariates based on a prespecified conceptual framework (Fig. 1 and see online supplementary material, Supplemental Fig. 1), using a direct acyclic graph approach^(27)^ informed by the published literature and data availability. Three multivariable models were used for each linear growth–bone outcome association: (i) adjustment for concurrent HAZ (i.e. HAZ at 4 years); (ii) adjustment for the full set of confounders but not concurrent HAZ and; (iii) adjustment for all confounders and concurrent HAZ; final inferences were informed from results of the fully adjusted model.

A similar modelling approach was used to estimate the associations between age interval-specific conditional weight gain (cWAZ) and each bone outcome at 4 years of age. However, for weight gain models, four multivariable models were used: the first three models were the same as those used in the length gain models, and a fourth model adjusted for all confounders, concurrent HAZ and concurrent WAZ at 4 years. Concurrent HAZ and concurrent WAZ were included to investigate the extent to which associations of early-life growth and bone outcomes were explained by (*v*. independent of) the effect of early-life growth on concurrent HAZ/WAZ. In a *post hoc* analysis assessing the effect of early-life relative weight gain on bone outcomes, we adjusted for cLAZ at 24 months of age to determine whether the association of cWAZ between 12 and 24 months, and bone mass was confounded by increases in length during this time period.

### Imputation methods for missing data

In primary analysis, missing data for explanatory variables were imputed using multivariate imputation by chained equations (MICE). Multivariate imputation by chained equations is a flexible data imputation method that generates multiple predictions for each missing value using a series of regression models based on the observed values for variables included in the imputation model. Predicted values are imputed to create multiple iterations of a complete dataset^(28)^. Here, variables were assumed to be missing at random, whereby the probability of missing data is considered independent of unobserved data and can be explained by observed data^(29)^. To obtain estimates of the exposure–outcome relationship using multivariate imputation by chained equations, analysis was performed using each complete dataset and final effect estimates are averaged across all datasets^(28)^. In total, 2·8 % of LAZ and WAZ measurements were missing within the first 45 d of age, in addition to some length and weight measurements missing at the 6-, 12- and 24-month timepoints (see online supplementary material, Supplemental Table 1). Additionally, there were five missing data points for other household covariates. Therefore, missing conditional growth measures (cLAZ and cWAZ at each age interval) and household characteristics (asset index, household smoking status and presence of hand soap in the handwashing station) were imputed to maximise the sample size when modelling the relationship between the conditional growth measure and the bone outcomes.

As a sensitivity analysis, a complete case analysis was conducted involving only infants without missing data for any covariates. In addition, we performed a restricted analysis in which preterm infants were excluded, as well as a subgroup analysis stratified by sex.

All statistical analyses were conducted using STATA version 17·1 (StataCorp).

## Results

### Participant characteristics

Of the 599 participants with a completed DEXA scan, 296 (49 %) were boys and 303 (51 %) were girls. Participant socio-demographic characteristics are presented in Table 1.

**Table 1.** Infant and maternal characteristics in the present study cohort overall and stratified by child sex

Characteristics	Total cohort (*n* 599)		Boys (*n* 296)		Girls (*n* 303)	
	*n* or mean or median	% or sd or min, max	*n* or mean or median	% or sd or min, max	*n* or mean or median	% or sd or min, max
Infant characteristics						
Gestational age at birth (weeks), median (min, max)	39	32, 43	39	33, 43	39	32, 43
Preterm (<37 weeks), *n* (%)	50	8	26	9	24	8
Mode of delivery, *n* (%)						
Vaginal birth	281	47	135	46	146	48
Caesarean section	318	53	161	54	157	52
Infant anthropometry, mean ± sd						
Birth weight (g)*	2715	352	2777	363	2659	333
Length at birth (cm)†	47·4	2·0	47·8	1·9	47·0	2·0
WAZ at birth*	–1·20	0·89	–1·16	0·89	–1·24	0·89
LAZ at birth†	–0·91	1·04	–0·86	0·99	–0·96	1·08
EBF duration (weeks), median (min, max)‡	14	0, 26	14	0, 26	14	0, 26
At least one diarrhoeal episode, *n* (%)§	283	47	153	52	130	43
Maternal characteristics						
Age (years), median (min, max)	23	18, 40	23	18, 38	23	18, 40
Height (cm), mean ± sd	150·8	5·5	151·0	5·3	150·5	5·7
Level of education, *n* (%)						
No schooling	25	4	10	3	15	5
Primary incomplete	113	19	50	17	63	21
Primary complete	87	15	43	15	44	15
Secondary incomplete	251	42	129	44	122	40
Secondary complete or higher	123	21	64	22	59	19
Mid-gestation Hb concentration (g/l), mean ± sd	107	11	106	11	107	11
Vitamin D intervention group, *n* (%)						
Placebo	114	19	51	17	63	21
4200 IU/week	126	21	64	22	62	21
16 800 IU/week	120	20	63	21	57	19
28 000 IU/week	121	20	56	19	65	21
28 000 IU/week prenatal and postpartum	118	20	62	21	56	19
Household characteristics						
Asset index quintiles, *n* (%)||						
1 (lowest)	113	19	57	19	56	19
2	118	20	64	22	54	18
3	119	20	50	17	69	23
4	131	22	61	21	70	23
5 (highest)	116	19	64	22	52	17
Household smoking, *n* (%)¶	214	36	107	36	107	35
Treatment of drinking water, *n* (%)	265	44	132	45	133	44
Presence of soap at handwashing station, *n* (%)**	499	83	249	84	250	83

aBMD, areal bone mineral density; BMC, bone mineral content; EBF, exclusive breastfeeding; HAZ, height-for-age *z*-score; LAZ, length-for-age *z*-score; WAZ, weight-for-age *z*-score; TBLH, total body less head.

* *n* 427 for the entire cohort, *n* 203 for boys, and *n* 224 for girls.

† *n* 423 for the entire cohort, *n* 200 for boys, and *n* 223 for girls.

‡ The maximum duration of EBF was an artifact of the study design which truncated this data up to the first 26 weeks of life.

§ Presence of at least one diarrhoeal episode in the first 6 months of life (yes *v*. no).

|| *n* 597 for the entire cohort and *n* 301 for girls. Asset index quintile variable derived using principal component analysis as measure of indicators of socioeconomic status based on ownership of claimed household items.

¶ *n* 597 for the entire cohort and *n* 294 for boys.

** *n* 598 for the entire cohort and *n* 295 for boys.

### Relative linear growth from birth to 2 years and bone outcomes at 4 years of age

**Figure. 2:**
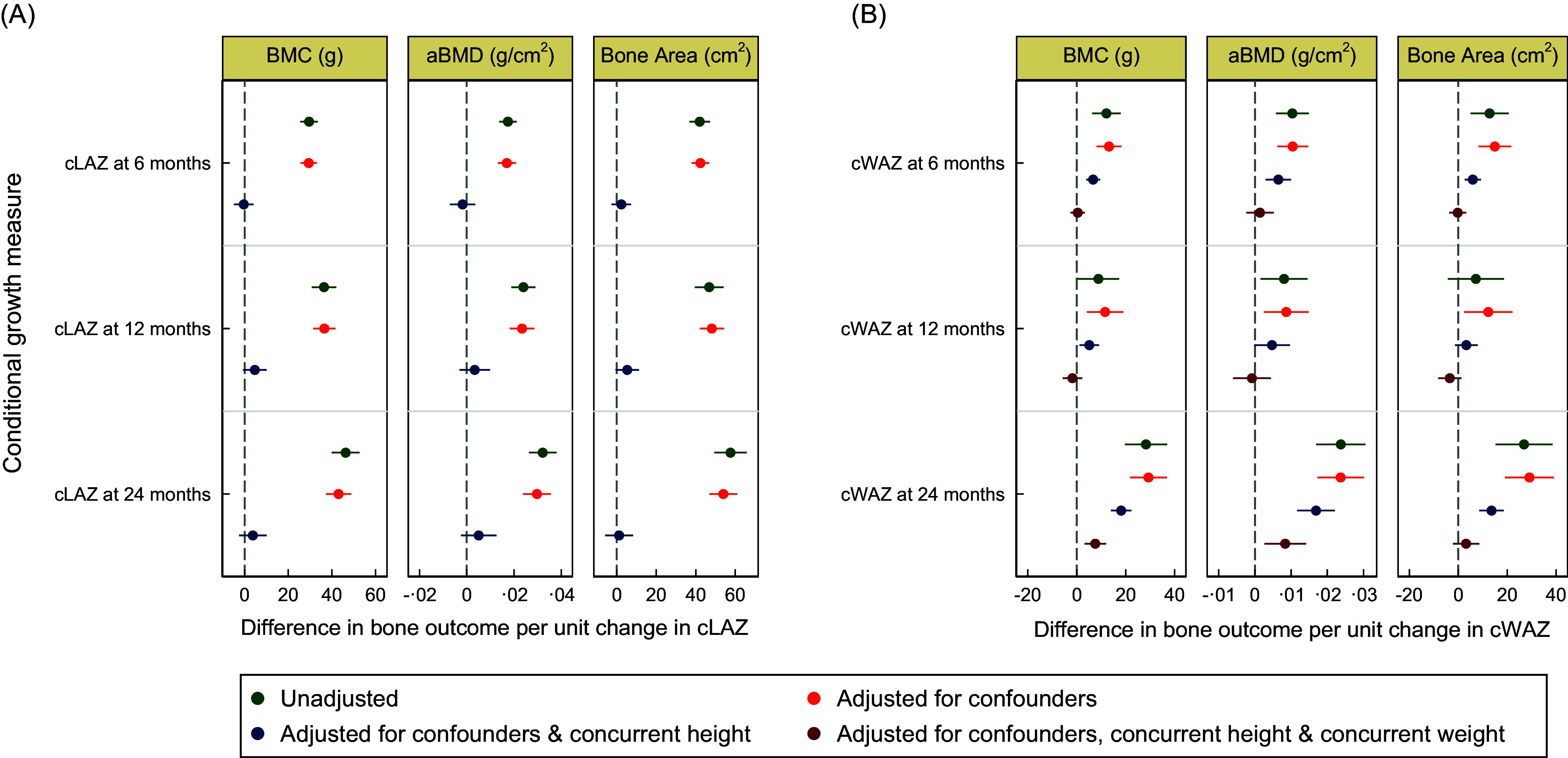
Associations of interval-specific conditional length gain (panel A) and weight gain (panel B) up to 2 years of age and bone outcomes at 4 years of age. Circles are point estimates and horizontal lines represent the 95 % confidence intervals. Unadjusted base models are represented in green. Multivariable models in orange were adjusted for confounders including: asset index quintiles, presence of hand soap at handwashing station, household smoking, LAZ within 45 d of birth, WAZ within 45 d of birth, maternal age (years), maternal height (cm), mid-gestation maternal Hb concentrations (g/l), gestational age at birth (weeks), maternal education category, vitamin D intervention group assigned at enrolment to the MDIG trial, treatment of drinking water, child sex, exclusive breastfeeding duration (weeks) and presence of diarrheal episodes in the first 6 months of life. Multivariable models adjusted for the aforementioned confounders and concurrent HAZ at 4 years of age are in blue. Multivariable models adjusted for the same confounders and concurrent HAZ at 4 years of age and concurrent WAZ at 4 years of age are in purple. Multiple imputation by chained equations (MICE) was used to impute missing data for explanatory variables. HAZ, height-for-age *z*-score; LAZ, length-for-age *z*-score; WAZ, weight-for-age *z*-score.

In unadjusted base models, cLAZ at 6, 12 and 24 months was positively associated with BMC, aBMD and bone area (Table 2 and Fig. 2). The magnitude of the effect estimates increased with age, with cLAZ at 24 months having the largest magnitude across all three bone measures. Including confounders (i.e. maternal, infant and household factors) in the model resulted in minimal changes to the effect estimates (Table 2). Concurrent HAZ at 4 years was strongly associated with all three bone outcomes, and estimates of associations of all early timepoints with bone parameters were substantially attenuated upon adjustment for HAZ at 4 years of age (Table 2). In the multivariable model including concurrent HAZ and other confounders, all estimates were further attenuated, and none remained statistically significant (Table 2).

**Table 2.** Associations between infant length growth from birth to 2 years of age and bone mineral content, bone mineral density and bone area at 4 years of age

	Base model			Multivariable model*			Adjusted for concurrent HAZ†			Multivariable model including concurrent HAZ‡		
	Difference in bone outcome§	95 % CI	*P*	Difference in bone outcome§	95 % CI	*P*	Difference in bone outcome§	95 % CI	*P*	Difference in bone outcome§	95 % CI	*P*
TBLH BMC (g)												
cLAZ (6 months)	29·5	25·5, 33·5	<0·001	29·4	25·7, 33·1	<0·001	2·3	–1·3, 5·8	0·22	–0·4	–4·9, 4·1	0·87
cLAZ (12 months)	36·4	30·8, 42·0	<0·001	36·5	31·4, 41·6	<0·001	7·7	3·1, 12·2	0·001	4·7	–0·7, 10·1	0·09
cLAZ (24 months)	46·3	40·0, 52·6	<0·001	43·0	37·2, 48·8	<0·001	8·8	3·4, 14·1	0·001	3·8	–2·5, 10·1	0·24
HAZ at 4 years	–		–	–		–	40·0	36·9, 43·2	<0·001	44·1	39·2, 49·0	<0·001
TBLH aBMD (g/cm^2^)												
cLAZ (6 months)	0·017	0·014, 0·021	<0·001	0·017	0·013, 0·021	<0·001	0·002	–0·002, 0·007	0·27	–0·002	–0·007, 0·004	0·51
cLAZ (12 months)	0·024	0·019, 0·029	<0·001	0·023	0·018, 0·029	<0·001	0·008	0·003, 0·014	0·003	0·003	–0·003, 0·010	0·31
cLAZ (24 months)	0·032	0·026, 0·038	<0·001	0·030	0·024, 0·036	<0·001	0·011	0·005, 0·018	<0·001	0·005	–0·003, 0·013	0·19
HAZ at 4 years	–		–	–		–	0·022	0·018, 0·026	<0·001	0·028	0·022, 0·034	<0·001
TBLH Bone Area (cm^2^)												
cLAZ (6 months)	42·0	36·8, 47·2	<0·001	42·3	37·9, 46·8	<0·001	2·4	–1·5, 6·3	0·23	2·3	–2·6, 7·3	0·36
cLAZ (12 months)	46·8	39·5, 54·1	<0·001	48·1	42·0, 54·2	<0·001	5·1	0·1, 10·1	0·04	5·3	–0·6, 11·3	0·08
cLAZ (24 months)	57·6	49·3, 65·8	<0·001	53·9	46·9, 61·0	<0·001	3·1	–2·8, 9·1	0·30	1·2	–5·9, 8·3	0·74
HAZ at 4 years	–		–	–		–	58·1	54·6, 61·6	<0·001	59·3	53·9, 64·7	<0·001

*n* 599 for all models. aBMD, areal bone mineral density; BMC, bone mineral content; cLAZ, conditional length-for-age *z*-score; HAZ, height-for-age *z*-score; TBLH, total body less head.

* Models adjusted for asset index quintile, presence of hand soap at handwashing station, household smoking, LAZ within 45 d of birth, WAZ within 45 d of birth, maternal age (years), maternal height (cm), mid-gestation maternal Hb concentrations (g/l), gestational age at birth (weeks), maternal education category, vitamin D intervention group assigned at enrolment to the MDIG trial, treatment of drinking water, child sex, exclusive breastfeeding duration (weeks) and presence of diarrheal episodes in the first 6 months of life.

† Models adjusted for HAZ at 4 years of age.

‡ Models adjusted for covariates in multivariable model and HAZ at 4 years of age.

§ Effect estimate represents the difference in bone outcome per 1 unit increase in cLAZ or HAZ.

### Relative weight gain from birth to 2 years and bone outcomes at 4 years of age

In the base models, the effect estimates for cWAZ at 24 months were more than double the magnitude of those for cWAZ at the 6-month or 12-month periods (Table 3 and Fig. 2). In multivariable models (adjusted for maternal, infant and household characteristics), cWAZ at all timepoints remained positively associated with bone outcomes with minimal impact on effect estimates (Table 3 and Fig. 2). Further adjustment for concurrent HAZ at 4 years in multivariable models resulted in attenuation of effect estimates, but inferences remained mostly unchanged (Table 3 and Fig. 2). In multivariable models that also included concurrent WAZ at 4 years, associations of cWAZ at 6- and 12 months with BMC and aBMD, and all associations between cWAZ and bone area were attenuated towards null and rendered non-significant (Table 3 and Fig. 2). Adjustment for concurrent WAZ also attenuated the positive associations between cWAZ at 24 months and BMC and aBMD, but these remained statistically significant (Table 3 and Fig. 2). In a *post hoc* analysis including adjustment for cLAZ at 24 months, cWAZ at 24 months remained significantly associated with BMC (β = 6·6 g; 95 % CI: 1·8, 11·4) and aBMD (β = 0·006 g/cm^2^; 95 % CI: 0·0002, 0·123) but not bone area (β = 3·5 cm^2^; 95 % CI: –2·4, 9·4).

**Table 3.** Associations between infant weight gain from birth to 2 years of age and bone mineral content, bone mineral density and bone area at 4 years of age

	Base model			Multivariable model*			Multivariable model including concurrent HAZ†			Multivariable model including concurrent HAZ & WAZ‡		
	Difference in bone outcome§	95 % CI	*P*	Difference in bone outcome§	95 % CI	*P*	Difference in bone outcome§	95 % CI	*P*	Difference in bone outcome§	95 % CI	*P*
TBLH BMC (g)												
cWAZ (6 months)	12·1	6·3, 17·9	<0·001	13·2	8·1, 18·3	<0·001	6·7	3·8, 9·5	<0·001	0·4	–2·5, 3·3	0·80
cWAZ (12 months)	8·9	0·4, 17·3	0·04	11·6	4·2, 19·0	0·002	5·1	1·1, 9·1	0·01	–1·8	–5·8, 2·2	0·38
cWAZ (24 months)	28·3	19·7, 36·9	<0·001	29·3	21·8, 36·9	<0·001	18·2	14·0, 22·3	<0·001	7·6	3·2, 12·0	0·001
HAZ at 4 years	–		–	–		–	43·1	40·8, 45·5	<0·001	27·2	23·3, 31·0	<0·001
WAZ at 4 years	–		–	–		–	–		–	19·8	15·8, 23·8	<0·001
TBLH aBMD (g/cm^2^)												
cWAZ (6 months)	0·010	0·006, 0·015	<0·001	0·010	0·006, 0·015	<0·001	0·007	0·003, 0·010	<0·001	0·001	–0·002, 0·005	0·46
cWAZ (12 months)	0·008	0·002, 0·015	0·02	0·009	0·002, 0·015	0·006	0·005	–0·0002, 0·010	0·06	–0·001	–0·006, 0·004	0·76
cWAZ (24 months)	0·024	0·017, 0·031	<0·001	0·024	0·017, 0·030	<0·001	0·017	0·012, 0·022	<0·001	0·008	0·003, 0·014	0·004
HAZ at 4 years	–		–	–		–	0·026	0·023, 0·029	<0·001	0·013	0·008, 0·018	<0·001
WAZ at 4 years	–		–	–		–	–		–	0·016	0·011, 0·021	<0·001
TBLH bone area (cm^2^)												
cWAZ (6 months)	12·8	5·0, 20·6	0·001	15·0	8·3, 21·6	<0·001	5·9	2·7, 9·2	<0·001	–0·2	–3·7, 3·2	0·89
cWAZ (12 months)	7·2	–4·3, 18·6	0·22	12·3	2·4, 22·2	0·02	3·3	–1·4, 8·0	0·17	–3·5	–8·2, 1·3	0·15
cWAZ (24 months)	26·9	15·2, 38·5	<0·001	29·1	19·1, 39·1	<0·001	13·6	8·6, 18·6	<0·001	3·2	–2·2, 8·6	0·24
HAZ at 4 years	–		–	–		–	59·7	57·0, 62·5	<0·001	44·1	39·4, 48·8	<0·001
WAZ at 4 years	–		–	–		–	–		–	19·4	14·6, 24·2	<0·001

*n* 599 for all models. aBMD, areal bone mineral density; BMC, bone mineral content; cWAZ, conditional weight-for-age *z*-score; HAZ, height-for-age *z*-score; TBLH, total body less head; WAZ, weight-for-age *z*-score.

* Models adjusted for asset index quintile, presence of hand soap at handwashing station, household smoking, LAZ within 45 d of birth, WAZ within 45 d of birth, maternal age (years), maternal height (cm), mid-gestation maternal Hb concentrations (g/l), gestational age at birth (weeks), maternal education category, vitamin D intervention group assigned at enrolment to the MDIG trial, treatment of drinking water, child sex, exclusive breast-feeding duration (weeks) and presence of diarrheal episodes in the first 6 months of life.

† Models adjusted for covariates in multivariable model and HAZ at 4 years of age.

‡ Models adjusted for covariates in multivariable model and HAZ at 4 years of age and WAZ at 4 years of age.

§ Effect estimate represents the difference in bone outcome per 1 unit increase in cWAZ, HAZ or WAZ.

### Stratified and sensitivity analyses

In the length gain models, analyses by child sex yielded similar inferences among boys and girls for most of the models (see online supplementary material, Supplemental Table 2). Analyses by child sex in all the weight gain models revealed similar patterns of associations and inferences, with only minor variation in the magnitude of effect sizes between boys and girls (see online supplementary material, Supplemental Table 3). Inferences from analyses restricted to term-born infants (i.e. ≥37 weeks) were consistent with the primary analysis for both length and weight gain models (see online supplementary material, Supplemental Tables 4 and 5); as observed in the primary analyses, associations of cWAZ at 24 months and bone mass remained significant (see online supplementary material, Supplemental Table 5). Lastly, in comparison to primary analysis using multivariate imputation by chained equations, similar inferences were obtained in sensitivity analyses restricted to participants without missing data for LAZ or WAZ within the first 2 years of life and/or any confounding variables included in multivariable models (see online supplementary material, Supplemental Tables 6 and 7).

## Discussion

In this cohort of children in Dhaka, Bangladesh, relative gains in both length and weight within the first 2 years of life were associated with increased bone mass and bone area at 4 years of age, with a greater magnitude of association for the latest age interval (12–24 months). However, the associations between early-life length gain and later bone outcomes were almost entirely explained by concurrent HAZ at 4 years, which was strongly associated with bone mass. The findings are consistent with previous longitudinal studies of associations between early-life growth and later bone mass in pediatric populations in both the Netherlands^(5)^ and the UK^(3)^, and studies of childhood growth and bone mass in adulthood in Finland^(30)^, India^(31)^ and Brazil^(32)^. In the context of the present study, in which postnatal linear growth faltering is pervasive, the results suggest that while interventions that promote linear growth from birth to 2 years of age may be expected to increase bone mass in proportion to height gain, there is no specific age interval in which bone mineral accrual is likely to be particularly sensitive to such interventions.

Faster relative weight gain within the first 2 years of life was associated with increased BMC, aBMD and bone area at 4 years of age, which was also consistent with prior studies^(4,5)^. Even after adjustment for concurrent WAZ, relative weight gain in the second year of life (12–24 months) remained significantly associated with higher BMC and aBMD, although, unlike previous findings from the Netherlands^(5)^, a similarly robust association with bone area was not observed. Endochondral ossification leading to bone elongation and mineral deposition is likely to be the primary mechanism that explains the associations of height and bone mass^(33)^, whereas the association of fat mass with bone mass is likely explained by mechanical loading that promotes appositional bone growth^(33,34)^. A study in the UK^(33)^ highlighted that the independent associations of height and weight gain with bone mass accrual, and their distinct underlying mechanisms of effect, makes it challenging to predict the effect of socio-economic factors on bone health; for example, whereas higher social position was associated with increased linear height and greater bone mass and size, higher social position was also associated with lower fat mass which was in turn associated with a lower bone mass. The relationship between relative weight gain in the 12–24-month interval and bone mass observed in the present study was unlikely to be explained by endochondral ossification, which couples bone lengthening to bone mineralisation, because the association was independent of length gains. During the second year of life, most children are ambulatory and therefore weight bearing, so it is likely that ponderal growth (gains in weight and adiposity) contributed to bone mineralisation by loading effects on the skeleton^(14,35)^. Increased weight supports both appositional bone formation and endocortical deposition by exerting mechanical stress on the skeleton at weight-bearing sites^(33,34)^. Given that we did not observe a significant association of relative weight gain with bone area, the primary mechanism supporting increased bone mass may therefore have been endocortical remodelling (which may not involve substantial changes in bone area); alternatively, it is possible that the association between appositional bone growth and bone area was too small to detect within the limits of the sample size of this study. Nonetheless, the findings indicate that the second year of a life may be a sensitive period in which changes in weight can affect the accumulation of bone mass through mechanisms other than bone elongation.

This study had several strengths. In extension of findings from high-income settings^(3,5)^, we examined the relationship between early-life growth and bone mass in a population in which linear growth faltering, undernutrition and low bone mass are common. Our findings are, therefore, particularly relevant for guiding future efforts targeting whole-population shifts in growth parameters. The conditional modelling approach facilitated examination of correlated growth measures in a single model, which enables identification of specific age periods in which interventions would be expected to have the most benefit on later bone mass, independent of growth in earlier or later age windows. Lastly, multiple imputation was used to address bias associated with missing data that is common in longitudinal studies, and reliability of the inferences was supported by complete-case analysis.

An important limitation of this study was that the role of physical activity was not investigated. Numerous studies have shown that physical activity influences skeletal integrity and bone mass^(36–38)^, and it likely contributed to the between-child variations and age-related differences in bone outcomes^(39)^. Height may be associated with gross motor activity of young children^(39)^, and therefore, physical activity may partially mediate the relationship between height and bone mass; however, there is also the potential for confounding relationships, given that height, bone mass and opportunities for physical activity are all likely to be related to socio-economic status. Similarly, while we accounted for the potential confounding of early-life infant feeding patterns, we lacked dietary intake data beyond the first 6 months of age; it is possible that inclusion of such data may have increased the precision of the effect estimates. Although DEXA is the gold-standard method for bone mass assessment in paediatrics^(40)^, it produces a two-dimensional estimate of a three-dimensional structure, which can lead to an overestimation of aBMD in larger bones and thereby potentially weaken the associations between body size and aBMD^(41,42)^. Finally, it is important to acknowledge that study participants were from a trial cohort recruited within one area of Dhaka, who were enrolled based on willingness and availability to participate in further follow-up studies. We have previously reported that socio-demographic characteristics were similar for MDIG trial participants and the subset of participants of the BONUSKids study included in the present analyses^(16)^; however, we cannot exclude the possibility of attrition bias and we are uncertain about the generalisability of our findings to the broader population of children in Dhaka or children residing in comparable settings.

## Conclusion

In a population with a high prevalence of linear growth faltering, relative gains in length in the first 2 years of life were associated with bone mass and bone area at 4 years of age, but there were no sensitive time periods when the associations were independent of concurrent HAZ at 4 years of age. Conversely, increased relative weight gain between the ages of 12–24 months may improve bone mass independently of linear growth or attained height and weight. However, any potential benefits of interventions that promote weight gain would need to be carefully balanced against the possible risks of excess weight to avoid unintended contributions to the double burden of malnutrition^(43)^.

## Supporting information

Gias et al. supplementary material Gias et al. supplementary material

## Supplementary material

For supplementary material accompanying this paper visit https://doi.org/10.1017/S1368980024002301
