# Supplementary material for: Associations of linear growth and weight gain in the first 2 years with bone mass at 4 years of age in children in Dhaka, Bangladesh
Source: Public Health Nutr. 2024 Nov 22;27(1):e265. doi: 10.1017/S1368980024002301 (PMC11705024; doi:10.1017/S1368980024002301)
Supplement: Gias et al. supplementary material [file S1368980024002301sup001.pdf]

Associations of linear growth and weight gain in the first 2 years with bone mass at 4 years of age in children in Dhaka, Bangladesh

Online Supplementary Material

Aug 1, 2024

Table of Contents

Supplemental Figure 1. Conceptual framework. 1

Supplemental Table 1. Missing anthropometric and household characteristics data at each timepoint 2

Supplemental Table 2. Associations between infant relative length gain from birth to 2 years of age and bone mineral content, density and area at 4 years of age, by child sex. 3

Supplemental Table 3. Associations between infant relative weight gain from birth to 2 years of age and bone mineral content, density and area at 4 years of age, by child sex. 5

Supplemental Table 4. Associations between infant relative length gain from birth to 2 years of age and bone mineral content, density and bone area at 4 years of age among term-born (≥37 weeks) infants compared to the full cohort. 7

Supplemental Table 5. Associations between infant relative weight gain from birth to 2 years of age and bone mineral content, density and area at 4 years of age among term-born (≥37 weeks) infants compared to the full cohort. 8

Supplemental Table 6. Associations between infant relative length gain from birth to 2 years of age and bone mineral content, density and area at 4 years of age, comparing MICE and complete case models. 9

Supplemental Table 7. Associations between infant relative weight gain from birth to 2 years of age and bone mineral content, density and area at 4 years of age, comparing MICE and complete case models. 10

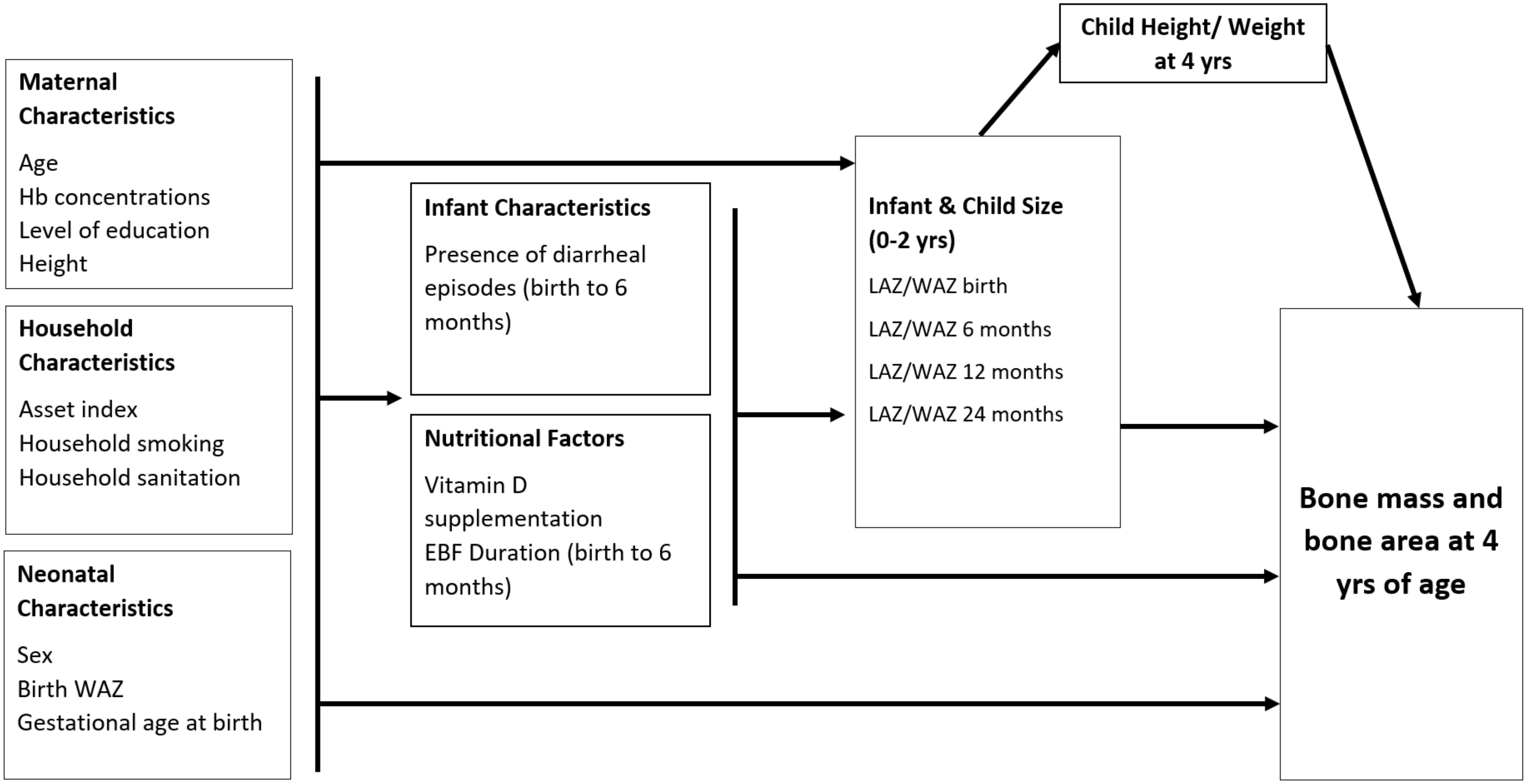

Supplemental Figure 1. Conceptual framework.

Figure shows hypothesized relationships between covariates included in analyses of the associations between linear growth, weight gain with bone mass and bone area at 4 years of age where maternal, household, neonatal, infant, and nutritional factors were hypothesized confounders of the association between early-life linear and ponderal growth on bone outcomes at 4 years of age; concurrent height and concurrent weight at 4 years of age were hypothesized as mediators of the relationship between early-life growth and bone mass at 4 years of age. EBF, exclusive breastfeeding; LAZ, length-for-age z-score; Hb, hemoglobin; WAZ, weight-for-age z-score.

Supplemental Table 1. Missing anthropometric and household characteristics data at each timepoint[a].

Variable	*N* Missing	% Missing
Earliest LAZ (<45 days of age)	17	2.8
cLAZ at 6 months	23	3.8
cLAZ at 12 months		4.0
cLAZ at 24 months	2	0.3
Earliest WAZ (<45 days of age)	17	2.8
cWAZ at 6 months	23	3.8
cWAZ at 12 months		4.5
cWAZ at 24 months	5	0.8
Asset index	2	0.3
Presence of hand soap in handwashing station	1	0.2
Household smoking	2	0.3

LAZ, length-for-age z-score; cLAZ, conditional LAZ; WAZ, weight-for-age z-score; cWAZ, conditional WAZ

[a]Missing variables identified at each timepoint of interest within an overall cohort sample size of 599. In primary analysis examining the association of growth within the first 2 years of life and bone outcomes at 4 years, missing data were imputed using multivariate imputation by chained equations (MICE).

Supplemental Table 2. Associations between infant relative length gain from birth to 2 years of age and bone mineral content, density and area at 4 years of age, by child sex.

	**Unadjusted model**		**Multivariable model[a]**		**Adjusted for concurrent HAZ[b]**		**Multivariable model including concurrent HAZ[c]**	
	Difference in bone outcome (95% CI)[d]	*P*	Difference in bone outcome (95% CI)[d]	*P*	Difference in bone outcome (95% CI)[d]	*P*	Difference in bone outcome (95% CI)[d]	*P*
**Boys (*n*=296)**								
**TBLH BMC (g)**								
cLAZ (6 months)	32.1 (26.6, 37.7)	<0.001	29.7 (24.5, 35.0)	<0.001	3.2 (-2.2, 8.6)	0.24	0.8 (-5.7, 7.2)	0.81
cLAZ (12 months)	35.9 (28.6, 43.3)	<0.001	33.9 (26.9, 40.9)	<0.001	7.3 (0.9, 13.8)	0.03	4.0 (-3.6, 11.6)	0.30
cLAZ (24 months)	48.2 (39.4, 57.0)	<0.001	47.5 (39.4, 55.7)	<0.001	13.9 (6.3, 21.6)	<0.001	9.0 (-0.2, 18.1)	0.06
HAZ at 4 years	–	–	–	–	38.6 (33.8, 43.3)	<0.001	42.0 (35.0, 48.9)	<0.001
**TBLH aBMD (g/cm²)**								
cLAZ (6 months)	0.019 (0.014, 0.024)	<0.001	0.017 (0.012, 0.023)	<0.001	0.004 (-0.003, 0.010)	0.28	0.001 (-0.007, 0.008)	0.82
cLAZ (12 months)	0.023 (0.017, 0.030)	<0.001	0.020 (0.013, 0.027)	<0.001	0.008 (0.001, 0.016)	0.03	0.003 (-0.006, 0.012)	0.49
cLAZ (24 months)	0.035 (0.027, 0.044)	<0.001	0.033 (0.025, 0.041)	<0.001	0.017 (0.008, 0.026)	<0.001	0.011 (0.0002, 0.022)	0.05
HAZ at 4 years	–	–	–	–	0.021 (0.015, 0.026)	<0.001	0.024 (0.016, 0.032)	<0.001
**TBLH Bone Area (cm²)**								
cLAZ (6 months)	44.8 (37.6, 52.1)	<0.001	42.0 (35.4, 48.7)	<0.001	1.9 (-4.2, 7.9)	0.55	1.1 (-6.4, 8.6)	0.77
cLAZ (12 months)	45.6 (35.9, 55.3)	<0.001	46.3 (37.4, 55.2)	<0.001	3.1 (-4.1, 10.3)	0.40	4.1 (-4.8, 12.9)	0.37
cLAZ (24 months)	56.9 (45.4, 68.5)	<0.001	59.2 (48.9, 69.5)	<0.001	5.9 (-2.7, 14.6)	0.18	4.8 (-6.0, 15.6)	0.39
HAZ at 4 years	–	–	–	–	57.4 (52.1, 62.8)	<0.001	59.3 (51.1, 67.4)	<0.001
**Girls (*n*=303)**								
**TBLH BMC (g)**								
cLAZ (6 months)	28.4 (22.7, 34.0)	<0.001	29.5 (24.1, 35.0)	<0.001	2.5 (-2.3, 7.4)	0.31	-1.5 (-8.1, 5.1)	0.66
cLAZ (12 months)	39.8 (31.3, 48.4)	<0.001	39.1 (31.3, 46.9)	<0.001	10.3 (3.6, 16.9)	0.003	5.7 (-2.4, 13.8)	0.17
cLAZ (24 months)	43.3 (34.3, 52.2)	<0.001	36.9 (28.3, 45.4)	<0.001	3.8 (-3.6, 11.3)	0.31	-1.4 (-10.3, 7.6)	0.76
HAZ at 4 years	–	–	–	–	40.6 (36.2, 45.0)	<0.001	45.6 (38.5, 52.7)	<0.001
**TBLH aBMD (g/cm²)**								
cLAZ (6 months)	0.017 (0.012, 0.022)	<0.001	0.017 (0.011, 0.022)	<0.001	0.003 (-0.003, 0.009)	0.38	-0.004 (-0.012, 0.004)	0.30
cLAZ (12 months)	0.027 (0.019, 0.035)	<0.001	0.026 (0.018, 0.034)	<0.001	0.011 (0.002, 0.019)	0.01	0.003 (-0.006, 0.013)	0.48
cLAZ (24 months)	0.028 (0.020, 0.036)	<0.001	0.025 (0.016, 0.034)	<0.001	0.006 (-0.003, 0.015)	0.18	-0.001 (-0.012, 0.010)	0.85
HAZ at 4 years	–	–	–	–	0.022 (0.017, 0.028)	<0.001	0.031 (0.022, 0.039)	<0.001
**TBLH Bone Area (cm²)**								
cLAZ (6 months)	40.8 (33.5, 48.1)	<0.001	43.3 (36.9, 49.6)	<0.001	3.6 (-1.6, 8.9)	0.17	3.3 (-3.8, 10.4)	0.36
cLAZ (12 months)	51.4 (40.5, 62.3)	<0.001	50.9 (41.9, 60.0)	<0.001	8.9 (1.7, 16.0)	0.02	7.9 (-0.8, 16.6)	0.07
cLAZ (24 months)	56.8 (45.2, 68.4)	<0.001	47.5 (37.4, 57.6)	<0.001	0.2 (-8.0, 8.4)	0.97	-1.7 (-11.5, 8.1)	0.74
HAZ at 4 years	–	–	–	–	58.3 (53.5, 63.1)	<0.001	58.7 (51.0, 66.5)	<0.001

aBMD, areal bone mineral density; BMC, bone mineral content; cLAZ, conditional length-for-age z-score; HAZ, height-for-age z-score; TBLH, total body less head.

[a]Adjusted for asset index quintiles, presence of hand soap at handwashing station, household smoking, LAZ within 45 days of birth, WAZ within 45 days of birth, maternal age (years), maternal height (cm), mid-gestation maternal hemoglobin concentrations (g/L), gestational age at birth (weeks), maternal education category, vitamin D intervention group assigned at enrolment to the MDIG trial, treatment of drinking water, child sex, exclusive breastfeeding duration (weeks), and presence of diarrheal episodes in the first 6 months of life.

[b]Adjusted for concurrent HAZ at 4 years of age.

[c]Adjusted for covariates in multivariable model and concurrent HAZ at 4 years of age..

[d] Effect estimate represents the difference in bone outcome per 1 unit increase in cLAZ or HAZ.

Supplemental Table 3. Associations between infant relative weight gain from birth to 2 years of age and bone mineral content, density and area at 4 years of age, by child sex.

	Unadjusted model		Multivariable model[a]		Multivariable model including concurrent HAZ[b]		Multivariable model including concurrent HAZ & WAZ[c]	
	Difference in bone outcome (95% CI)[d]	*P*	Difference in bone outcome (95% CI)[d]	*P*	Difference in bone outcome (95% CI)[d]	*P*	Difference in bone outcome (95% CI)[d]	*P*
**Boys (*n*=296)**								
**TBLH BMC (g)**								
cWAZ (6 months)	12.0 (4.0, 20.1)	0.004	14.4 (7.5, 21.3)	<0.001	7.2 (3.2, 11.1)	<0.001	0.3 (-3.9, 4.4)	0.89
cWAZ (12 months)	4.0 (-7.8, 15.9)	0.50	3.7 (-6.6, 14.0)	0.48	3.7 (-2.0, 9.5)	0.20	-3.9 (-9.7, 2.0)	0.19
cWAZ (24 months)	34.7 (22.9, 46.5)	<0.001	34.8 (24.9, 44.8)	<0.001	19.2 (13.5, 25.0)	<0.001	6.8 (0.5, 13.1)	0.03
HAZ at 4 years	–	–	–	–	41.8 (38.4, 45.2)	<0.001	24.4 (18.7, 30.1)	<0.001
WAZ at 4 years	–	–	–	–	–	–	21.5 (15.6, 27.4)	<0.001
**TBLH aBMD (g/cm^2)**								
cWAZ (6 months)	0.012 (0.006, 0.018)	<0.001	0.013 (0.007, 0.018)	<0.001	0.008 (0.004, 0.013)	<0.001	0.003 (-0.002, 0.008)	0.26
cWAZ (12 months)	0.005 (-0.004, 0.015)	0.25	0.003 (-0.006, 0.012)	0.51	0.003 (-0.004, 0.010)	0.42	-0.003 (-0.011, 0.005)	0.44
cWAZ (24 months)	0.026 (0.017, 0.036)	<0.001	0.025 (0.017, 0.033)	<0.001	0.016 (0.009, 0.023)	<0.001	0.006 (-0.002, 0.014)	0.13
HAZ at 4 years	–	–	–	–	0.024 (0.020, 0.029)	<0.001	0.011 (0.003, 0.018)	0.004
WAZ at 4 years	–	–	–	–	–	–	0.017 (0.009, 0.024)	<0.001
**TBLH Bone Area (cm^2)**								
cWAZ (6 months)	10.3 (-0.6, 21.1)	0.06	14.7 (5.4, 24.1)	0.002	4.6 (-0.3, 9.4)	0.06	-2.3 (-7.5, 2.9)	0.38
cWAZ (12 months)	-1.2 (-17.3, 14.8)	0.88	1.4 (-12.6, 15.5)	0.84	1.5 (-5.5, 8.5)	0.68	-6.1 (-13.4, 1.1)	0.10
cWAZ (24 months)	37.0 (21.1, 52.9)	<0.001	38.8 (25.3, 52.4)	<0.001	16.8 (9.7, 24.0)	<0.001	4.4 (-3.7, 12.5)	0.29
HAZ at 4 years	–	–	–	–	59.0 (54.7, 63.2)	<0.001	41.5 (34.3, 48.8)	<0.001
WAZ at 4 years	–	–	–	–	–	–	21.5 (14.0, 29.1)	<0.001
**Girls (*n*=303)**								
**TBLH BMC (g)**								
cWAZ (6 months)	12.5 (4.2, 20.8)	0.003	12.5 (4.9, 20.1)	0.001	5.7 (1.5, 9.9)	0.007	-0.7 (-4.9, 3.6)	0.76
cWAZ (12 months)	13.9 (2.4, 25.4)	0.02	17.7 (6.9, 28.5)	0.001	6.4 (0.6, 12.2)	0.03	-0.1 (-5.8, 5.6)	0.98
cWAZ (24 months)	19.6 (6.8, 32.4)	0.003	21.6 (9.7, 33.4)	<0.001	17.5 (11.1, 23.9)	<0.001	7.7 (1.2, 14.2)	0.02
HAZ at 4 years	–	–	–	–	43.6 (40.3, 47.0)	<0.001	28.2 (22.9, 33.6)	<0.001
WAZ at 4 years	–	–	–	–	–	–	19.4 (13.9, 24.9)	<0.001
**TBLH aBMD (g/cm^2)**								
cWAZ (6 months)	0.009 (0.002, 0.016)	0.008	0.008 (0.001, 0.014)	0.02	0.004 (-0.002, 0.009)	0.17	-0.002 (-0.008, 0.003)	0.45
cWAZ (12 months)	0.011 (0.002, 0.020)	0.02	0.014 (0.005, 0.023)	0.003	0.007 (-0.001, 0.014)	0.07	0.001 (-0.007, 0.008)	0.85
cWAZ (24 months)	0.020 (0.010, 0.030)	<0.001	0.021 (0.011, 0.031)	<0.001	0.018 (0.010, 0.026)	<0.001	0.009 (0.001, 0.018)	0.03
HAZ at 4 years	–	–	–	–	0.027 (0.023, 0.031)	<0.001	0.013 (0.006, 0.020)	<0.001
WAZ at 4 years	–	–	–	–	–	–	0.018 (0.010, 0.025)	<0.001

	**Unadjusted model**		**Multivariable model[a]**		**Multivariable model including concurrent HAZ[b]**		**Multivariable model including concurrent HAZ & WAZ[c]**	
	Difference in bone outcome (95% CI)[d]	*P*	Difference in bone outcome (95% CI)[d]	*P*	Difference in bone outcome (95% CI)[d]	*P*	Difference in bone outcome (95% CI)[d]	*P*
**TBLH Bone Area (cm^2)**								
cWAZ (6 months)	15.9 (4.7, 27.0)	0.005	17.2 (7.2, 27.1)	0.001	7.8 (3.1, 12.6)	0.001	2.5 (-2.6, 7.6)	0.33
cWAZ (12 months)	15.5 (-0.1, 31.0)	0.05	20.2 (6.0, 34.3)	0.005	4.7 (-2.1, 11.4)	0.17	-0.8 (-7.6, 6.0)	0.82
cWAZ (24 months)	13.6 (-3.7, 30.8)	0.12	16.7 (1.2, 32.2)	0.04	11.1 (3.6, 18.5)	0.004	2.9 (-5.0, 10.7)	0.47
HAZ at 4 years	–	–	–	–	59.8 (55.9, 63.7)	<0.001	46.9 (40.5, 53.3)	<0.001
WAZ at 4 years	–	–	–	–	–	–	16.3 (9.7, 22.8)	<0.001

aBMD, areal bone mineral density; BMC, bone mineral content; cWAZ, conditional weight-for-age z-score; HAZ, height-for-age z-score; TBLH, total body less head.

[a]Adjusted models for asset index quintiles, presence of hand soap at handwashing station, household smoking, LAZ within 45 days of birth, WAZ within 45 days of birth, maternal age (years), maternal height (cm), mid-gestation maternal hemoglobin concentrations (g/L), gestational age at birth (weeks), maternal education category, vitamin D intervention group assigned at enrolment to the MDIG trial, treatment of drinking water, child sex, exclusive breastfeeding duration (weeks), and presence of diarrheal episodes in the first 6 months of life; [b]Adjusted for covariates in multivariable model and concurrent HAZ at 4 years of age; [c]Adjusted for covariates in multivariable model and concurrent HAZ at 4 years of age and concurrent WAZ at 4 years of age; [d] Effect estimate represents the difference in bone outcome per 1 unit increase in cLAZ or HAZ.

Supplemental Table 4. Associations between infant relative length gain from birth to 2 years of age and bone mineral content, density and bone area at 4 years of age among term-born (≥37 weeks) infants compared to the full cohort.

	**Multivariable model (Full cohort, *n*=599)[a]**		**Multivariable model (Term born, *n*=549)[a]**		**Multivariable model including concurrent HAZ (Full cohort, *n*=599)[b]**		**Multivariable model including concurrent HAZ (Term born, *n*=549)[b]**	
	Difference in bone outcome (95% CI)	*P*	Difference in bone outcome (95% CI)	*P*	Difference in bone outcome (95% CI)	*P*	Difference in bone outcome (95% CI)	*P*
**TBLH BMC (g)**								
cLAZ (6 months)	29.4 (25.7, 33.1)	<0.001	29.3 (25.5, 33.2)	<0.001	-0.4 (-4.9, 4.1)	0.87	-0.1 (-4.7, 4.4)	0.95
cLAZ (12 months)	36.5 (31.4, 41.6)	<0.001	37.5 (32.1, 42.9)	<0.001	4.7 (-0.7, 10.1)	0.09	5.4 (-0.3, 11.1)	0.06
cLAZ (24 months)	43.0 (37.2, 48.8)	<0.001	43.3 (37.3, 49.3)	<0.001	3.8 (-2.5, 10.1)	0.24	4.3 (-2.3, 10.8)	0.20
HAZ at 4 years	–	–	–	–	44.1 (39.2, 49.0)	<0.001	43.7 (38.6, 48.8)	<0.001
**TBLH aBMD (g/cm^2)**								
cLAZ (6 months)	0.017 (0.013, 0.021)	<0.001	0.017 (0.013, 0.021)	<0.001	-0.002 (-0.007, 0.004)	0.51	-0.002 (-0.007, 0.004)	0.55
cLAZ (12 months)	0.023 (0.018, 0.029)	<0.001	0.024 (0.018, 0.029)	<0.001	0.003 (-0.003, 0.010)	0.31	0.004 (-0.003, 0.010)	0.32
cLAZ (24 months)	0.030 (0.024, 0.036)	<0.001	0.030 (0.024, 0.036)	<0.001	0.005 (-0.003, 0.013)	0.19	0.005 (-0.003, 0.013)	0.19
HAZ at 4 years	–	–	–	–	0.028 (0.022, 0.034)	<0.001	0.027 (0.021, 0.034)	<0.001
**TBLH Bone Area (cm^2)**								
cLAZ (6 months)	42.3 (37.9, 46.8)	<0.001	42.6 (37.9, 47.2)	<0.001	2.3 (-2.6, 7.3)	0.36	2.9 (-2.2, 8.0)	0.27
cLAZ (12 months)	48.1 (42.0, 54.2)	<0.001	49.7 (43.2, 56.3)	<0.001	5.3 (-0.6, 11.3)	0.08	6.5 (0.3, 12.8)	0.04
cLAZ (24 months)	53.9 (46.9, 61.0)	<0.001	54.4 (47.2, 61.7)	<0.001	1.2 (-5.9, 8.3)	0.74	1.9 (-5.4, 9.2)	0.61
HAZ at 4 years	–	–	–	–	59.3 (53.9, 64.7)	<0.001	58.9 (53.2, 64.5)	<0.001

aBMD, areal bone mineral density; BMC, bone mineral content; cLAZ, conditional length-for-age z-score; HAZ, height-for-age z-score; TBLH, total body less head.

[a]Adjusted models for asset index quintiles, presence of hand soap at handwashing station, household smoking, LAZ within 45 days of birth, WAZ within 45 days of birth, maternal age (years), maternal height (cm), mid-gestation maternal hemoglobin concentrations (g/L), gestational age at birth, maternal education category, vitamin D intervention group assigned at enrolment to the MDIG trial, treatment of drinking water, sex, exclusive breastfeeding duration, and presence of diarrheal episodes in the first 6 months of life.

[b]Adjusted models for covariates in multivariable model and concurrent HAZ at 4 years of age.

Supplemental Table 5. Associations between infant relative weight gain from birth to 2 years of age and bone mineral content, density and area at 4 years of age among term-born (≥37 weeks) infants compared to the full cohort.

	**Multivariable model (Full cohort, *n*=599)**[a]		**Multivariable model (Term born, *n*=549)**[a]		**Multivariable model including concurrent HAZ & WAZ (Full cohort, *n*=599)**[b]		**Multivariable model including concurrent HAZ & WAZ (Term born, *n*=549)**[b]	
	Difference in bone outcome (95% CI)	*P*	Difference in bone outcome (95% CI)	*P*	Difference in bone outcome (95% CI)	*P*	Difference in bone outcome (95% CI)	*P*
**TBLH BMC (g)**								
cWAZ (6 months)	13.2 (8.1, 18.3)	<0.001	12.9 (7.7, 18.1)	<0.001	0.4 (-2.5, 3.3)	0.80	0.7 (-2.3, 3.7)	0.64
cWAZ (12 months)	11.6 (4.2, 19.0)	0.002	12.1 (4.4, 19.8)	0.002	-1.8 (-5.8, 2.2)	0.38	-0.9 (-5.2, 3.3)	0.66
cWAZ (24 months)	29.3 (21.8, 36.9)	<0.001	30.0 (22.0, 38.0)	<0.001	7.6 (3.2, 12.0)	0.001	7.9 (3.2, 12.6)	0.001
HAZ at 4 years	–	–	–	–	27.2 (23.3, 31.0)	<0.001	27.8 (23.7, 31.8)	<0.001
WAZ at 4 years	–	–	–	–	19.8 (15.8, 23.8)	<0.001	18.9 (14.8, 23.0)	<0.001
**TBLH aBMD (g/cm^2)**								
cWAZ (6 months)	0.010 (0.006, 0.015)	<0.001	0.010 (0.006, 0.015)	<0.001	0.001 (-0.002, 0.005)	0.46	0.002 (-0.002, 0.006)	0.36
cWAZ (12 months)	0.009 (0.002, 0.015)	0.006	0.009 (0.002, 0.015)	0.009	-0.001 (-0.006, 0.004)	0.76	-0.001 (-0.006, 0.005)	0.82
cWAZ (24 months)	0.024 (0.017, 0.030)	<0.001	0.025 (0.018, 0.032)	<0.001	0.008 (0.003, 0.014)	0.004	0.009 (0.003, 0.016)	0.003
HAZ at 4 years	–	–	–	–	0.013 (0.008, 0.018)	<0.001	0.013 (0.008, 0.019)	<0.001
WAZ at 4 years	–	–	–	–	0.016 (0.011, 0.021)	<0.001	0.016 (0.010, 0.021)	<0.001
**TBLH Bone Area (cm^2)**								
cWAZ (6 months)	15.0 (8.3, 21.6)	<0.001	14.2 (7.3, 21.2)	<0.001	-0.2 (-3.7, 3.2)	0.89	-0.3 (-3.8, 3.2)	0.86
cWAZ (12 months)	12.3 (2.4, 22.2)	0.02	13.3 (3.0, 23.6)	0.01	-3.5 (-8.2, 1.3)	0.15	-2.2 (-7.2, 2.9)	0.40
cWAZ (24 months)	29.1 (19.1, 39.1)	<0.001	29.2 (18.5, 39.8)	<0.001	3.2 (-2.2, 8.6)	0.24	2.7 (-2.9, 8.4)	0.34
HAZ at 4 years	–	–	–	–	44.1 (39.4, 48.8)	<0.001	45.4 (40.6, 50.2)	<0.001
WAZ at 4 years	–	–	–	–	19.4 (14.6, 24.2)	<0.001	18.2 (13.3, 23.2)	<0.001

aBMD, areal bone mineral density; BMC, bone mineral content; cWAZ, conditional weight-for-age z-score; HAZ, height-for-age z-score; TBLH, total body less head.

[a]Adjusted models for asset index quintiles, presence of hand soap at handwashing station, household smoking, LAZ within 45 days of birth, WAZ within 45 days of birth, maternal age (years), maternal height (cm), mid-gestation maternal hemoglobin concentrations (g/L), gestational age at birth, maternal education category, vitamin D intervention group assigned at enrolment to the MDIG trial, treatment of drinking water, sex, exclusive breastfeeding duration, and presence of diarrheal episodes in the first 6 months of life.

[b]Adjusted models for covariates in multivariable model, concurrent HAZ at 4 years of age, and concurrent WAZ at 4 years of age.

Supplemental Table 6. Associations between infant relative length gain from birth to 2 years of age and bone mineral content, density and area at 4 years of age, comparing MICE and complete case models.

	Multivariable model (MICE model, *n*=599)[a]		Multivariable model (Complete case, *n*=554)[a]		Multivariable model including concurrent HAZ (MICE model, *n*=599)[b]		Multivariable model including concurrent HAZ (Complete case, *n*=554)[b]	
	Difference in bone outcome (95% CI)	*P*	Difference in bone outcome (95% CI)	*P*	Difference in bone outcome (95% CI)	*P*	Difference in bone outcome (95% CI)	*P*
**TBLH BMC (g)**								
cLAZ (6 months)	29.4 (25.7, 33.1)	<0.001	28.7 (25.0, 32.5)	<0.001	-0.4 (-4.9, 4.1)	0.87	-2.4 (-7.0, 2.1)	0.30
cLAZ (12 months)	36.5 (31.4, 41.6)	<0.001	37.1 (31.9, 42.2)	<0.001	4.7 (-0.7, 10.1)	0.09	3.2 (-2.4, 8.7)	0.27
cLAZ (24 months)	43.0 (37.2, 48.8)	<0.001	42.4 (36.5, 48.3)	<0.001	3.8 (-2.5, 10.1)	0.24	1.5 (-5.0, 8.1)	0.65
HAZ at 4 years	–	–	–	–	44.1 (39.2, 49.0)	<0.001	46.5 (41.3, 51.6)	<0.001
**TBLH aBMD (g/cm^2)**								
cLAZ (6 months)	0.017 (0.013, 0.021)	<0.001	0.017 (0.013, 0.021)	<0.001	-0.002 (-0.007, 0.004)	0.51	-0.004 (-0.009, 0.002)	0.18
cLAZ (12 months)	0.023 (0.018, 0.029)	<0.001	0.024 (0.019, 0.029)	<0.001	0.003 (-0.003, 0.010)	0.31	0.002 (-0.005, 0.008)	0.62
cLAZ (24 months)	0.030 (0.024, 0.036)	<0.001	0.030 (0.024, 0.036)	<0.001	0.005 (-0.003, 0.013)	0.19	0.003 (-0.005, 0.011)	0.49
HAZ at 4 years	–	–	–	–	0.028 (0.022, 0.034)	<0.001	0.031 (0.024, 0.037)	<0.001
**TBLH Bone Area (cm^2)**								
cLAZ (6 months)	42.3 (37.9, 46.8)	<0.001	41.5 (36.9, 46.0)	<0.001	2.3 (-2.6, 7.3)	0.36	0.4 (-4.8, 5.6)	0.88
cLAZ (12 months)	48.1 (42.0, 54.2)	<0.001	49.0 (42.7, 55.2)	<0.001	5.3 (-0.6, 11.3)	0.08	4.3 (-2.1, 10.7)	0.18
cLAZ (24 months)	53.9 (46.9, 61.0)	<0.001	52.4 (45.2, 59.6)	<0.001	1.2 (-5.9, 8.3)	0.74	-1.4 (-8.9, 6.0)	0.71
HAZ at 4 years	–	–	–	–	59.3 (53.9, 64.7)	<0.001	61.2 (55.3, 67.1)	<0.001

aBMD, areal bone mineral density; BMC, bone mineral content; cLAZ, conditional length-for-age z-score; HAZ, height-for-age z-score; MICE, multiple imputation by chained equations; TBLH, total body less head.

[a]Adjusted models for asset index quintiles, presence of hand soap at handwashing station, household smoking, LAZ within 45 days of birth, WAZ within 45 days of birth, maternal age (years), maternal height (cm), mid-gestation maternal hemoglobin concentrations (g/L), gestational age at birth, maternal education category, vitamin D intervention group assigned at enrolment to the MDIG trial, treatment of drinking water, sex, exclusive breastfeeding duration, and presence of diarrheal episodes in the first 6 months of life.

[b]Adjusted models for covariates in multivariable model and concurrent HAZ at 4 years of age.

Supplemental Table 7. Associations between infant relative weight gain from birth to 2 years of age and bone mineral content, density and area at 4 years of age, comparing MICE and complete case models.

	**Multivariable model (MICE model, *n*=599)[a]**		**Multivariable model (Complete case, *n*=551)[a]**		**Multivariable model including concurrent HAZ & WAZ (MICE model, *n*=599)[b]**		**Multivariable model including concurrent HAZ & WAZ (Complete case, *n*=551)[b]**	
	Difference in bone outcome (95% CI)	*P*	Difference in bone outcome (95% CI)	*P*	Difference in bone outcome (95% CI)	*P*	Difference in bone outcome (95% CI)	*P*
**TBLH BMC (g)**								
cWAZ (6 months)	13.2 (8.1, 18.3)	<0.001	13.3 (8.3, 18.4)	<0.001	0.4 (-2.5, 3.3)	0.80	-0.1 (-3.0, 2.8)	0.92
cWAZ (12 months)	11.6 (4.2, 19.0)	0.002	12.3 (5.0, 19.6)	0.001	-1.8 (-5.8, 2.2)	0.38	-2.1 (-6.0, 1.9)	0.31
cWAZ (24 months)	29.3 (21.8, 36.9)	<0.001	28.7 (20.9, 36.4)	<0.001	7.6 (3.2, 12.0)	0.001	6.8 (2.4, 11.3)	0.003
HAZ at 4 years	–	–	–	–	27.2 (23.3, 31.0)	<0.001	26.7 (22.7, 30.7)	<0.001
WAZ at 4 years	–	–	–	–	19.8 (15.8, 23.8)	<0.001	20.7 (16.6, 24.7)	<0.001
**TBLH aBMD (g/cm^2)**								
cWAZ (6 months)	0.010 (0.006, 0.015)	<0.001	0.011 (0.006, 0.015)	<0.001	0.001 (-0.002, 0.005)	0.46	0.001 (-0.003, 0.005)	0.64
cWAZ (12 months)	0.009 (0.002, 0.015)	0.006	0.009 (0.003, 0.016)	0.004	-0.001 (-0.006, 0.004)	0.76	-0.001 (-0.006, 0.004)	0.68
cWAZ (24 months)	0.024 (0.017, 0.030)	<0.001	0.023 (0.017, 0.030)	<0.001	0.008 (0.003, 0.014)	0.004	0.007 (0.001, 0.013)	0.02
HAZ at 4 years	–	–	–	–	0.013 (0.008, 0.018)	<0.001	0.013 (0.008, 0.018)	<0.001
WAZ at 4 years	–	–	–	–	0.016 (0.011, 0.021)	<0.001	0.017 (0.012, 0.023)	<0.001
**TBLH Bone Area (cm^2)**								
cWAZ (6 months)	15.0 (8.3, 21.6)	<0.001	15.1 (8.4, 21.8)	<0.001	-0.2 (-3.7, 3.2)	0.89	-0.5 (-4.1, 3.1)	0.79
cWAZ (12 months)	12.3 (2.4, 22.2)	0.02	13.2 (3.5, 22.9)	0.008	-3.5 (-8.2, 1.3)	0.15	-3.5 (-8.5, 1.4)	0.16
cWAZ (24 months)	29.1 (19.1, 39.1)	<0.001	28.3 (18.0, 38.5)	<0.001	3.2 (-2.2, 8.6)	0.24	2.8 (-2.8, 8.4)	0.32
HAZ at 4 years	–	–	–	–	44.1 (39.4, 48.8)	<0.001	44.1 (39.2, 49.0)	<0.001
WAZ at 4 years	–	–	–	–	19.4 (14.6, 24.2)	<0.001	19.6 (14.6, 24.6)	<0.001

aBMD, areal bone mineral density; BMC, bone mineral content; cWAZ, conditional weight-for-age z-score; HAZ, height-for-age z-score; MICE, multiple imputation by chained equations; TBLH, total body less head.

[a]Adjusted models for asset index quintiles, presence of hand soap at handwashing station, household smoking, LAZ within 45 days of birth, WAZ within 45 days of birth, maternal age (years), maternal height (cm), mid-gestation maternal hemoglobin concentrations (g/L), gestational age at birth, maternal education category, vitamin D intervention group assigned at enrolment to the MDIG trial, treatment of drinking water, sex, exclusive breastfeeding duration, and presence of diarrheal episodes in the first 6 months of life.

[b]Adjusted models for covariates in multivariable model, concurrent HAZ at 4 years of age and concurrent WAZ at 4 years of age.
